# In Vitro and In Vivo Evaluations of Mesoporous Iron Particles for Iron Bioavailability

**DOI:** 10.3390/ijms20215291

**Published:** 2019-10-24

**Authors:** Jung-Feng Lin, Chau-Chung Wu, Yu-Jiun Liao, Subhaini Jakfar, Zi-Biao Tang, Jhewn-Kuang Chen, Feng-Huei Lin

**Affiliations:** 1 Department of Biomedical Engineering, National Taiwan University, Taipei 10617, Taiwan; D99548005@ntu.edu.tw (J.-F.L.); D05548020@ntu.edu.tw (S.J.); 2 Department of Internal Medicine (Cardiology Section), National Taiwan University Hospital, Taipei 10051, Taiwan; chauchungwu@ntu.edu.tw; 3 Department of Materials and Mineral Resources Engineering, National Taipei University of Technology, Taipei 10608, Taiwan; aw22451357@hotmail.com (Y.-J.L.); tptang87@gmail.com (Z.-B.T.); jkchen@ntut.edu.tw (J.-K.C.); 4 Institute of Biomedical Engineering and Nanomedicine, National Health Research Institutes, Miaoli 35053, Taiwan

**Keywords:** hemodialysis, iron deficiency anemia, mesoporous iron particle, hemoglobin

## Abstract

Chronic renal failure involving hemodialysis results in blood loss during filtration. Iron deficiency and iron deficiency anemia can result. A compensatory increase in iron dosage has many side effects including discomfort. Elemental iron is a highly-pure iron source, which reduces the frequency of dosages; the solubility decreases with increased particle size or pore size. In this study, synthesized mesoporous iron particles (MIPs) were used to relieve iron deficiency anemia. Their bioavailability was measured in vitro by a Caco-2 cell model and in vivo in iron-deficient rats. In vitro bioavailability of MIPs was examined by measuring ferritin content in the Caco-2 cell model. Iron uptake of MIPs was significantly higher than commercial iron particles, which were less porous. In vivo bioavailability of MIPs was examined by measuring body weight gain and red blood cell-related parameters, compared with the bioavailability of standard drug ferrous sulfate in iron-deficient anemic rats. Finally, average hemoglobin content and hemoglobin regeneration efficiency were significantly higher in anemic rats supplemented with commercial iron particles, compared to anemic controls. In the 28-day oral toxicity test, MIPs were not significantly toxic to rat physiology or tissue histopathology. Thus, MIPs may allow effective recovery of hemoglobin in iron deficiency anemia.

## 1. Introduction

Iron is an abundant element and a biologically essential component of human cells. Humans require iron for the synthesis of oxygen transport proteins, both hemoglobin and myoglobin, and for the formation of heme enzymes and other iron-containing enzymes involved in electron transfer and oxidation-reduction [1]. Hemodialysis (HD) patients experience the sustained loss of iron due to poor food intake, renal dialysis during hemorrhage and hemolysis, bleeding in the gastrointestinal tract, and blood loss during testing. The estimated annual blood loss for HD patients is approximately 3 g [2]. Blood loss is the main cause of iron deficiency anemia (IDA). IDA can present with symptoms that include skin paleness, fatigue, dyspnea, and headaches [3]. In the hospital, most HD patients require iron supplements. Iron supplementation plays an important role in reducing renal failure.

In most patients, iron deficiency is treated by supplementation of iron in the diet or directly. Dietary iron consists of heme iron and non-heme iron. The main source of heme iron for animal tissues is hemoglobin and myoglobin, while non-heme iron comes mainly from vegetables [4]. Over-ingestion can be a weight burden and wasteful economically. As an alternative, the use of intravenous (IV) iron and oral iron supplements have been explored for hospitalized IDA patients. Oral iron should be the first-line treatment for IDA, with IV delivery of iron used only when intestinal absorption is known to be impaired, such as in cases of gastrostomy [5]. In contrast, oral iron supplementation is an inexpensive, safe, and effective means of increasing hemoglobin levels and restoring iron stores to prevent iron deficiency. 

Oral iron supplements include elemental iron, ferrous iron, and ferric iron. For optimal absorption, oral iron supplements must dissolve rapidly in acidic gastric secretions. Intestinal absorption requires iron to be delivered to the duodenum and proximal jejunum at an acidic pH in an ionic form and the divalent (ferrous) state. Ferric iron is less soluble in the stomach. Its metabolism requires the presence of ferric reductase present on the membranes of duodenal microvilli. The enzyme catalyzes the conversion of ferric iron to ferrous iron [6]. Ferrous sulfate (FS) is the most common oral iron supplement in IDA therapy. However, it often causes gastrointestinal side effects, including nausea, constipation, and diarrhea [7]. Elemental iron comprises reduced iron, electrolytic iron, and carbonyl iron that can provide more than 99% of the elemental form. Relative to FS, which provides only 20% iron source, elemental iron can provide 100% of the iron required by patients. 

Supplementation with elemental iron can avoid the gastrointestinal side effects. The success of this strategy depends on ionization of the elemental iron in the presence of acid to release ferrous iron for absorption [8]. The bioavailability of elemental iron particles is inversely related to particle size and shape. The decreased size of element iron particles increases the solubility and bioavailability of iron [9,10,11,12]. Bioavailability of iron is greater for spherical particles because of their greater surface area and dissolution rate. The solubility of iron particles gradually increases with decreasing granularity and high surface area, which result in easier dissolution during digestion and much higher bioavailability.

In order to increase the dissolution of iron in gastric acid, we prepared high porosity and surface area mesoporous iron particles (MIPs). Wet chemistry techniques, including sol–gel and surfactant emulsion processes, have been combined with porogens to fabricate macroporous and mesoporous particles [13]. In addition, the porous structure can be increased by temperature regulation [14] or addition to the porogens, such as water, NaCl [15], or polyvinylpyrrolidone (PVP) [16]. In this study, we used polyvinyl alcohol (PVA) as the porogen, because it is soluble in water and slightly soluble in ethanol [17]. As well, PVA is a non-toxic and biocompatible polymer that has been widely used in biomedical engineering and pharmaceutical technology applications [18].

In this study, chemically reduced iron particles [19] and PVA were used to synthesize MIPs. The crystal structure of the synthesized MIPs was confirmed using X-ray diffractometry (XRD). Additionally, scanning electron microscopy (SEM) and transmission electron microscopy (TEM) were used to determine the morphology and chemical composition of the synthesized particles. Zeta analysis was used to measure the synthesized particle size. Surface area and pore size distribution were measured based on the Brunauer, Emmett, and Teller (BET) theory. The porosity distribution of the synthesized particles was measured using a mercury porosimeter. The influence of the MIPs on cell viability was determined using the water-soluble tetrazolium (WST-1) test. In vitro, immunohistochemistry staining was used to determine tight junction structure during the differentiation of Caco-2 cell monolayers. Cellular iron uptake was determined by direct measurements using standard ferrous sulfate, commercial iron particles, and the synthesized MIPs by measuring ferritin as the intracellular stored form of iron. In vivo, the safety of MIPs supplied orally in repeated doses was assessed for 28 days using Wistar rats. Finally, in vivo testing was also performed using the AOAC rat hemoglobin regeneration method to assess the bioavailability of the MIPs.

## 2. Results

### 2.1. Characterization of MIPs

**Figure 1:**
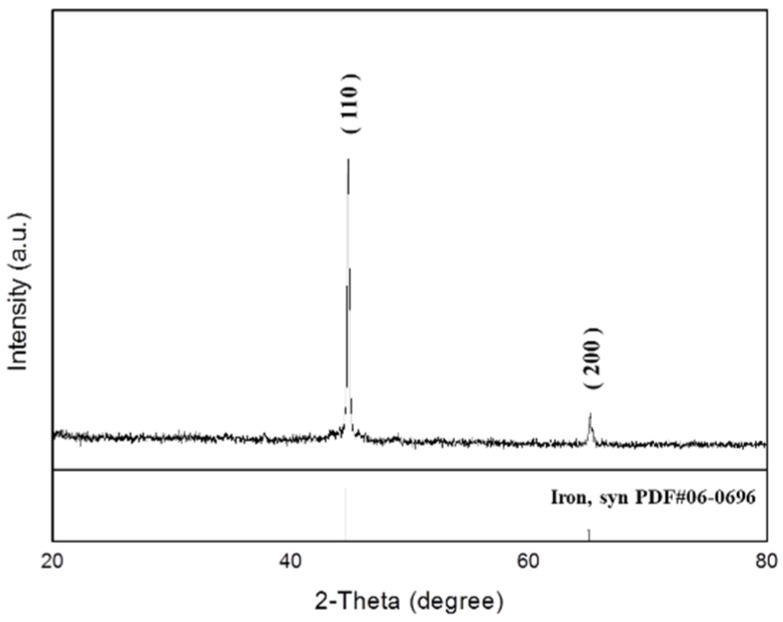
X-ray diffractometry (XRD) of synthesized mesoporous iron particles.

The typical XRD diffraction pattern of freshly synthesized MIPs is shown in Figure 1. The XRD pattern was recorded using Cu Kα radiation at a 2θ range of 20° to 80°. All the characteristic peaks matched the standard JCPD 06-0696 pattern. The two major peaks at 44.67 and 65.02° corresponded to the diffracted beam of the planes (110) and (200) from the crystal lattice of the MIPs.

**Figure 2:**
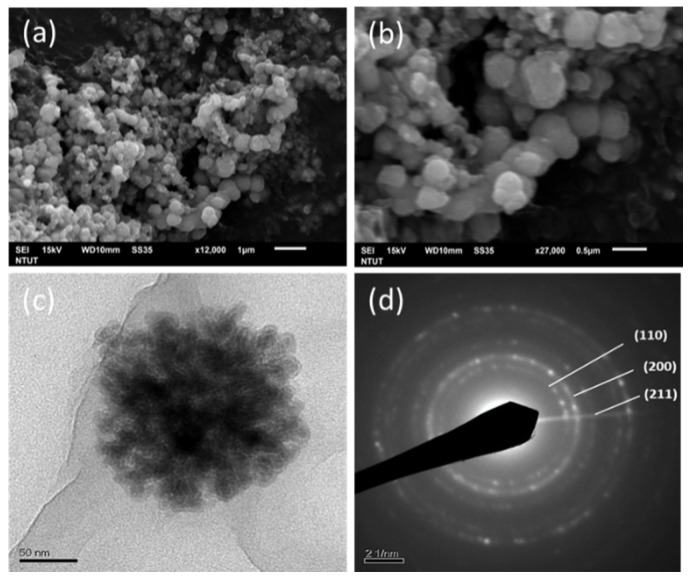
Scanning electron microscopy (SEM) of mesoporous iron particles at magnifications of 1200× (**a**) and 2700× (**b**), TEM appearance at 6000× (**c**), and diffraction pattern (**d**).

**Figure 3:**
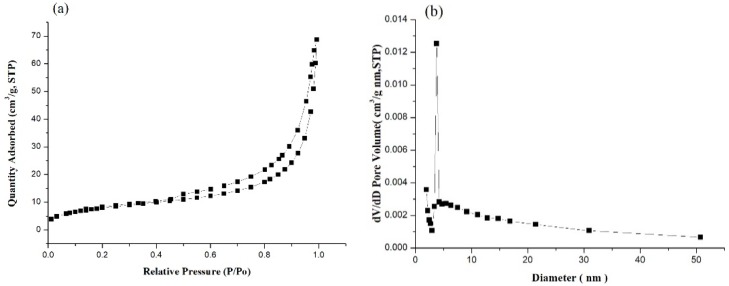
Nitrogen adsorption-desorption isotherm (**a**) and pore size distribution curve (**b**) of mesoporous iron particles.

The surface morphology of MIPs was characterized by SEM (Figure 2a,b). MIPs were typically spherical. The structure and diffraction pattern of the material were examined by TEM as shown in Figure 2c,d, respectively. MIPs formed nanoaggregates with a particle size ranging from 200 to 300 nm. The selected area diffraction pattern of MIPs revealed a pattern of concentric rings from the interior to the exterior, representing the crystal planes of (110), (200), and (211) which corresponds to the anatase phase. The particle size was precisely measured using a Zetasizer (Figure 3). The article size was 338.3 nm, which agreed with the SEM observations.

The porosity of MIPs was determined using the nitrogen adsorption/desorption curve and the pore size distribution in Figure 3. Figure 3a displays a representative nitrogen adsorption/desorption isotherm curve. The BET surface area was 28.37 m^2^/g, the Langmuir surface area was 45.77 m^2^/g, and the pore volume was 0.093 cm^3^/g. Figure 3b depicts the pore size distribution. The distribution was concentrated between 2 and 50 nm, with an average pore diameter of 13.14 nm. Mercury porosimeter analysis revealed an MIP porosity of 78.55%.

**Figure 4:**
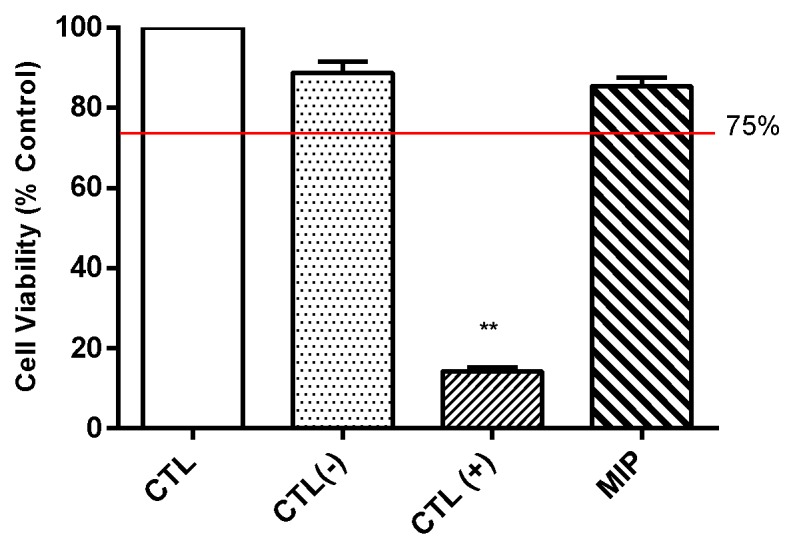
Biocompatibility of mesoporous iron particles. CTL: control. CTL(−): negative control, Aluminum oxide. CTL(+): positive control, zinc diethyldithiocarbamate. MIP: mesoporous iron particles. Compared with CTL group, the cell viability more than 75% (Red line) indicated not potential toxicity. ** *p* < 0.01 compared with control group, *n* = 6.

The influence of MIPs on cell viability determined using the WST-1 test is depicted in Figure 4. Cell viability was 14.23% ± 0.88% in the positive control group, 88.67% ± 2.64% in the negative control group, 100% ± 3.86% in the control group, and 85.42% ± 1.87% in the MIP group. The cell viability of the control group was 100% at baseline. Each experiment was repeated five times. According to the regulations of ISO 10993 [20], a cell viability exceeding 75% indicates no potential toxicity. Thus, the MIPs were not toxic to normal cells.

### 2.2. In Vitro Caco-2 Monolayer Model of MIPs

**Figure 5:**
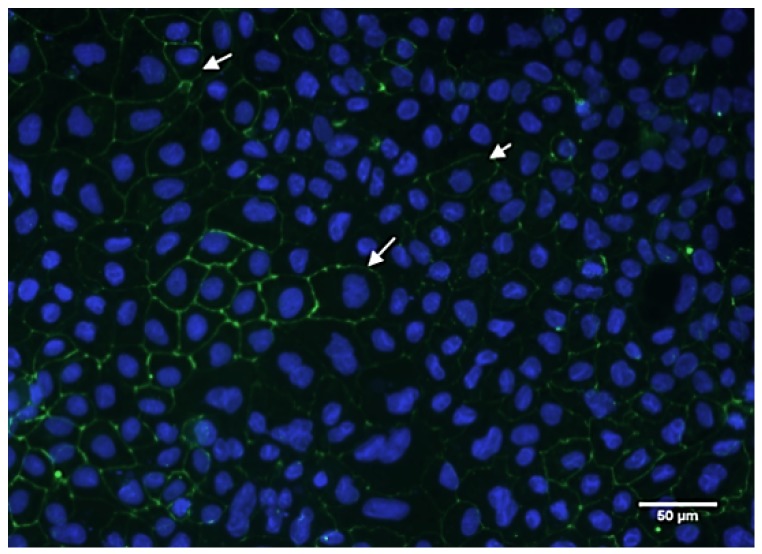
Immunofluorescence of differentiating human Caco-2 cells. Green fluorescence indicates ZO-1 (white arrow) and blue fluorescence indicates nuclei of cells. Scale bar: 50 μm.

The immunohistochemistry results are shown in Figure 5. Tight junction proteins were clearly observed in differentiating Caco-2 cells. Indicated by the white arrow, the zonula occludens-1 (ZO-1) protein was stained green and the nuclei of cells stained blue. The immunofluorescence staining revealed the production of ZO-1 protein between Cacao-2 cells after 21 days of culture. Cell monolayers had a tight junction structure, confirming their suitability for iron uptake experiments.

**Figure 6:**
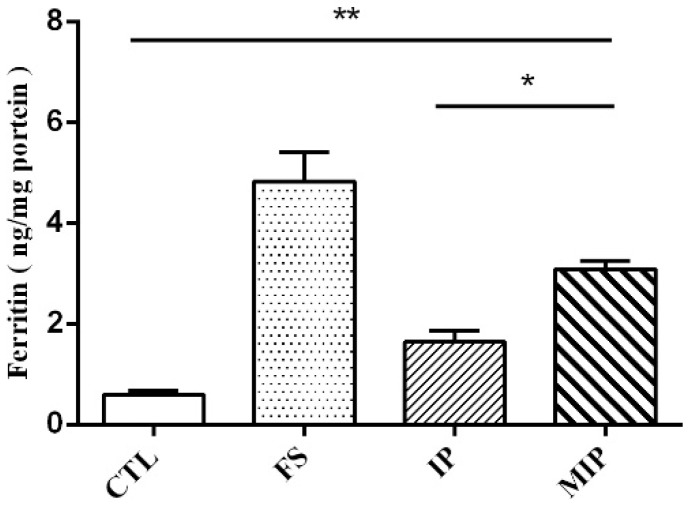
Ferritin levels in mono-cultured Caco-2 cells treated with iron. CTL: control; FS: FeSO_4_·7H_2_O; IP: commercial iron particles; MIP: mesoporous iron particles. All data are presented as mean ± SD (*n* = 3). The result s compared with control group, * *p* < 0.05; ** *p* < 0.01.

Ferritin concentrations in Caco-2 cells treated with iron standard drug (FS), commercial iron particles (IP), and the synthesized MIPs were examined (Figure 6). Ferritin concentrations were 0.59 ng/mg protein (control group), 4.83 ng/mg protein (FS group), 1.63 ng/mg protein (IP), and 3.08 ng/mg protein (MIPs). The IP and MIPs are water-insoluble, which reduced their bioavailability compared to the water-soluble FS. However, compared with the two water-insoluble iron powders, the pore iron powder displayed a significant difference from the commercial iron powder. The findings indicated that increasing the porosity of the iron powder effectively increased iron bioavailability.

### 2.3. In Vivo Oral Subacute Toxicity of MIPs

**Figure 7:**
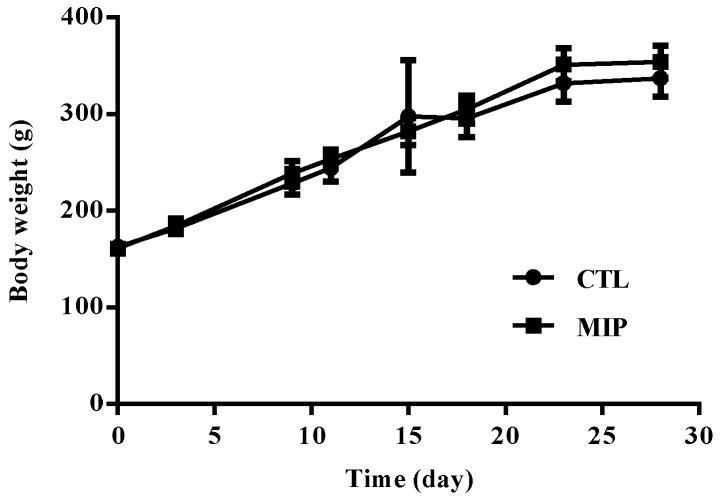
Body weight of rats after 28 day sub-aute oral toxicity testing with MIPs. CTL: control; MIP: mesoporous iron particles. All data are presented as mean ± SD (*n* = 10).

**Figure 8:**
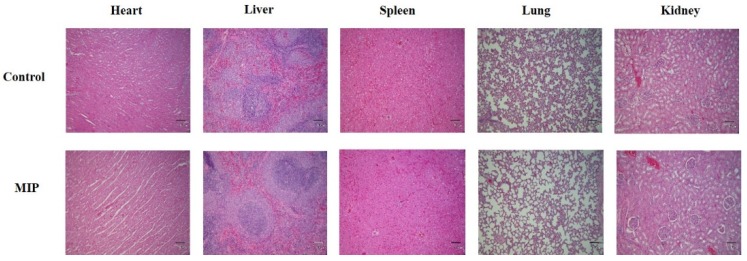
Hematoxylin and eosin (H&E) staining of rat tissues after 28 day subacute oral toxicity experiment with MIPs (40×; scale bar: 100 μm). CTL: control; MIP: mesoporous iron particles.

The change of the body weight of the experiment group of rats is presented in Figure 7. Oral subacute dosages of MIPs did not affect body weight in the 28 day experiment compared to the control groups. Before sacrifice, cardiac puncture was done to obtain blood for biochemical and whole blood analyses. The results were compared with the reference ranges [21]. The hematological value of the control and MIP groups are shown in Table 1. Repeated oral administration for 28 days results in hematological parameters in the standard range. Serum biochemical parameters in the 28 day subacute toxicity experiment were in standard range for serum biochemical parameters among the control and MIP groups (Table 2). Heart, liver, spleen, lung, and kidney tissue samples were fixed in 10% formalin in phosphate buffered saline (PBS) and cut into sections that were observed by light microscopy. The MIP particles evident in the 28 day subacute toxicity experiment are shown in Figure 8. Histological observations of heart, liver, spleen, lung, and kidneys did not reveal significate differences between the MIP and control groups. Hematoxylin and eosin (H&E) staining of the control and MIP groups are shown in Figure 8. The results of serum biochemistry, whole blood, and histological analyses indicated that MIPs did not cause obvious systemic toxicity.

### 2.4. Effect of MIPs on Iron Bioavailability and Oral Acute Toxicity in Rats

**Figure 9:**
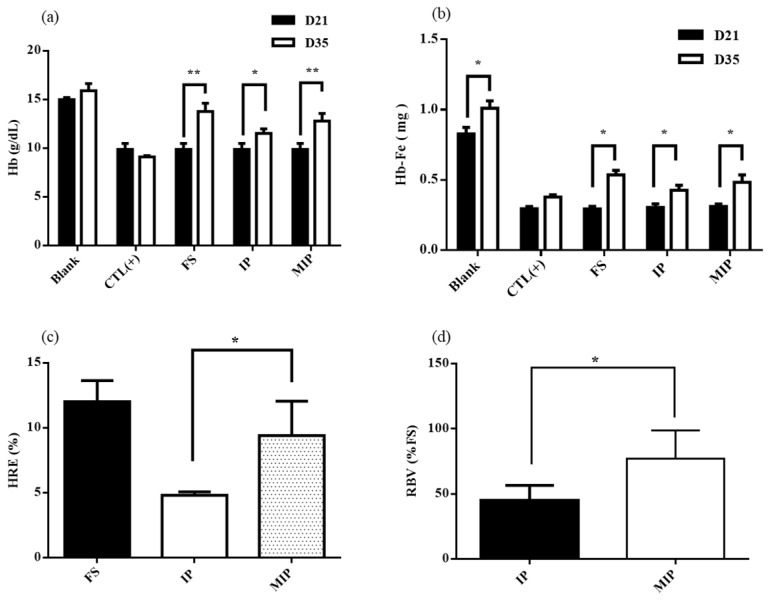
Hemoglobin and iron responses in the rat hemoglobin regeneration assay after 14 days: (**a**) Hb, (**b**) Hb-Fe, (**c**) HRE, and (**d**) RBV. Blank: Normal diet; CTL(+): AIN-93G diet; FS: AIN-93G + 24 mg/kg FeSO_4_·7H_2_O; IP: AIN-93G + 24 mg/kg commercial iron particles; MIP: AIN-93G + 24 mg/kg mesoporous iron particles. All data are presented as mean ± SD (*n* = 6). * *p* < 0.05; ** *p* < 0.01.

After the iron depletion period of 21 days in rats, the hemoglobin concentration was 14.97 ± 0.19 g/dL (blank group) and 9.87 ± 0.57 g/dL (AIN-93G group). The difference was significant. Rats receiving the AIN-93G feed were already anemic (Figure 9a). After repletion with iron supplements, the hemoglobin concentration gain was 0.9 ± 0.6 g/dL (blank group), –0.8 ± 0.1 g/dL (positive control group), 3.9 ± 0.7 g/dL (FS group), 1.5 ± 0.3 g/dL (IP group), and 2.9 ± 0.7 g/dL (MIPs group) (Figure 9a). After the repletion period, the hemoglobin (Hb) and hemoglobin iron (Hb-Fe) in the FS, IP, and MIP groups were significantly higher than the values at the time of iron depletion (Figure 9b). The effects of iron repletion on hemoglobin regeneration efficiency (HRE) and relative biological value (RBV) of the FS, IP, and MIP groups are shown in Figure 9c,d. After iron repletion, HRE of the FS, IP, and MIP groups were 12% ± 1.41%, 6.07% ± 1.57%, and 9.41% ± 2.41% respectively. RBVs of the IP and MIP groups were 49.82% ± 12.91% and 77.22% ± 19.77%. The MIP group showed the most significant change in RBV (*p* < 0.05).

**Figure 10:**
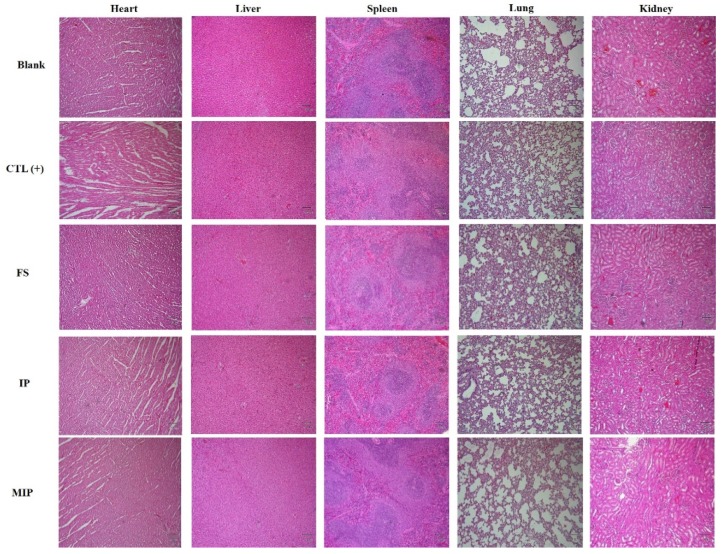
H&E staining of rat tissues after 14 day repletion in rats (40×; scale bar: 100 μm). Blank: normal diet; CTL(+): AIN-93G diet; FS: AIN-93G + 24 mg/kg FeSO_4_·7H_2_O; IP: AIN-93G + 24 mg/kg commercial iron particles; MIP: AIN-93G + 24 mg/kg mesoporous iron particles.

All the biochemical analysis and whole blood analysis results were compared with the reference ranges [21]. The hematological values of the blank, positive control, FS, IP, and MIP groups after iron repletion are shown in Table 3. For hematological parameters, repeated iron supplementation for the 14 day oral administration produced values that were mostly within the reference range. In addition to the positive control group, the values of the red blood cell correlation analysis of the rats was lower than the values other groups due to the continuous administration of iron-deficient feed. The serum biochemical parameters of the blank, positive control, FS, IP, and MIP groups after iron repletion are shown in Table 4. After the end of the repletion period, serum biochemical analysis of the rats revealed values within the reference range. H&E staining of the blank, positive control, FS, IP, and MIP groups are shown in Figure 10. Organs were fixed in 10% formalin in PBS and cut into sections that were observed by light microscopy. Histological observations of heart, liver, spleen, lung, and kidneys did not reveal significant differences.

## 3. Discussion

The oral delivery of iron salts to treat iron deficiency anemia has several shortcomings, which include inconvenience caused by increasing the dose and the number of doses of the iron salt, and the need for iron binder after consuming the iron salt. The resulting side effects include gastrointestinal discomfort. Efforts to improve the problems of current oral iron therapy have included providing a high content of elemental iron to reduce the dosage and reducing the side effects of elemental iron. However, to increase the bioavailability of oral elemental iron, the main prerequisite is the ability of elemental iron to dissolve in gastric acid to release iron ions, which are then absorbed by the intestinal mucosa into the body. The iron salt in the oral iron agent can quickly dissolve in an aqueous solution of gastric acid, while elemental iron is insoluble in aqueous solution. The solubility of elemental iron might be increased by reducing the particle size of the particles and increasing the surface area. Previous studies have indicated that particle size reduction is effective in improving bioavailability in vitro using Caco-2 cells. The spherical iron particles also have a high surface area, which can improve their bioavailability in vitro and in vivo. The orally consumed iron particles rapidly dissolved in the gastric juice in the stomach to form iron ions, which subsequently formed porous and spherical MIPs. The description of MIPs in iron deficiency anemia is novel.

MIPs were successfully synthesized by the modified borohydride reduction method. The crystal phase was identified and surface morphology was observed. In hexane, polysorbate 80 is nonionic surfactant and PVA as porogen that can be used to prepare iron particles. Upon the removal of PVA and nonionic surfactants, iron particles with medium-sized holes are formed. SEM and TEM observations revealed spherical shape of the particles (Figure 2). The nitrogen adsorption/desorption isotherm curve of the iron particles revealed the solidification of the nitrogen molecule between P/P0 = 0.4 and 1.0, due to condensation of the capillaries in the mesopores (Figure 3a). Hysteresis as the gas is desorbed is the typical type IV hole adsorption mode. Its hysteresis mode is H3, in which the iron particles of the pores are slit-like aggregates of particles [22]. The observations confirmed the mesoporous nature of the synthesized iron particles. This structure allows rapid entry of gastric acid into the particles and the release of iron ions. Compared with the surface area of commercial iron particles, which ranged 0.2 to 0.4 m^2^/g [23], the surface area of synthesized MIPs was much larger. The porosity and the spherical shape effectively increased the surface area of the MIPs, which in turn effectively increased dissolution of the particles in the stomach.

According to the ISO 10993 standards, MIPs were not toxic to L929 cells (Figure 4). Furthermore, in the 28-day oral feed toxicity test, the MIPs did not significantly detrimentally alter physiological parameters of rats (Figure 7 and Figure 8; and Table 1 and Table 2).

The Caco-2 cell model is widely used to assess the bioavailability of iron in vitro. During the differentiation of the cells into cells with properties of cells of the small intestine, iron can be digested by simulating the stomach and small intestine, and the iron content of the intracellular storage form of iron is measured [24]. In the Caco-2 cell iron experiment, the same dose of commercial iron particles and MIPs were digested using simulated gastric juice for 2 h. The particle size and surface area affected the solubility. An inverse relationship was described between particle size and iron bioavailability, and a linear relationship was observed between solubility and iron bioavailability [12]. The surface area of iron particles was more predictive of iron bioavailability [25]. Similarly, if iron particles were produced in the same production mode, smaller particle size was associated with increased bioavailability (Figure 6) of the water-soluble FS standard drug (RBV 100%), in which the porous iron particles have good ferritin generating capacity (RBV 63.8%). It is presumed that as the particle size becomes smaller, the porosity is increased to increase the surface area to in turn permit rapid dissolution in gastric acid.

The specific bioavailability can be determined by the AOAC rat hemoglobin repletion bioassay. The assay is based on the principle that iron deficiency anemia rats have the greatest absorption capacity for food iron, which is preferentially used to synthesize red blood cells. Presently, rats whose red blood cells (RBC), hemoglobin (Hb), and hematocrit (HCT) were lower than the standard value during the 21 day iron consumption period were all anemic (Table 5). After 14 days of feeding iron supplements, FS was used as the standard drug (RBV 100%), in which the porous iron particles (RBV 77%) were more bioavailable than the common commercial iron particles (RBV 50%). Compared to other commercial elements, carbonyl iron particles are spherical and have a high surface area; hence, their bioavailability in vitro is much greater than other particles (RBV 64%) [26] because of their comparatively rapid dissolution in an acidic solution [23]. The MIPs prepared in this study were spherical, similar to commercial iron particles, but were more porous. Thus, in the rat hemoglobin repletion bioassay, the MIPs displayed superior bioavailability than the carbonyl iron particles.

Iron overload induces organ damage in liver and other organ systems. The main cause of damage is due to the overproduction of reactive oxygen species in the presence of excess iron [27]. To examine the possible hepatotoxicity of MIPs, serum activities of aspartate aminotransferase and alanine aminotransferase were examined. The elevated serum activities of each hepatocyte enzyme indicates liver damage [28]. The blood biochemical results revealed similar levels of the enzymes during the dosing oral subacute toxicity and iron repletion period in all rats, indicating that the ingestion of MIP didn’t cause hepatotoxicity (Table 2 and Table 4). Therefore, ingestion of MIP at the daily dose of 24 mg/kg can increase the hemoglobin without deleterious effects in iron-deficient rats. The collective findings indicate that MIP does not have obvious systemic toxicity.

## 4. Materials and Methods

### 4.1. Preparation of MIPs

MIPs were prepared according to a previously reported method [19] with slight modifications using PVA as the porogen to create the porous structure. Briefly, iron pursuer solution was prepared by mixing 1.0 g PVA (363146; Sigma-Aldrich, St. Louis, MO, USA), 0.5 mL Tween-80 (P1754; Sigma-Aldrich, St. Louis, MO, USA), and 1.22 g FeCl_3_ (157740; Sigma-Aldrich, St. Louis, MO, USA) in distilled water 100 mL. The reduction solution was prepared by mixing 0.5 mL Tween-80 and 0.95 g NaBH_4_ (213462; Sigma-Aldrich, St. Louis, MO, USA) in 100 mL of distilled water. Both solutions were added drop-wise to the oil-phase n-Hexane (296090; Sigma-Aldrich, St. Louis, MO, USA) using a 25 G syringe during 30 min. The mixtures were heated at 70 °C to volatilize the hexane. The precipitate was collected by centrifugation and washed three times with ethanol and deionized water. All samples were stored in a −80 °C freezer, freeze-dried, and stored in a vacuum dryer oven.

### 4.2. Characterization of MIPs

The particles were mounted on the sample holder of a D2 phase X-ray powder diffraction meter (Bruker, Hamburg, Germany) for crystal structure identification. The XRD pattern was obtained at 40 kV and 30 mA with a 2θ range of 20° to 80° at a count time of 0.5 s for each step. The 2θ shift, lattice constant, d-spacing, and crystallinity were calculated based on the XRD pattern using MDI Jade 5.0 XRD equipped software (Materials Data Inc., Liverpool, CA).

The morphology of the synthesized particles was observed by SEM using a model JSM-7610F microscope (JEOL, Tokyo, Japan) with an accelerating voltage of 15 kV. The sample was suspended in ethanol. One drop of the colloidal solution was deposited on the SEM platform coated carbon tape and evaporated in an oven. The synthetic particles’ structure and diffraction pattern were analyzed by TEM using a model 2010F microscope (JEOL).

The specific surface area and the pore size distribution of the synthesized particles were determined by BET (Micromeritics ASAP 2020M, Micromeritics Instrument Corp. USA) using nitrogen (N_2_) gas adsorption analyzer. Specific surface areas and pore size distributions of the MIPs were calculated by the BET (Brunauer–Emmett–Teller) and BJH (Barrett–Joiner–Halenda) method.

The porosity of particles was determined by mercury porosimetry using an AutoPore^®^ IV 9520 (Japan). The particle size were detected under water by a Zetasizer Nano ZS90 (Malvern, Worcestershire, UK) operating at 10 to 70 °C (± 0.1 °C).

### 4.3. Cell Culture

L929 cells were purchased from Bio-resource Collection and Research Center (FIRDI, Hsin-Chu City, Taiwan). The cells were used for in vitro biocompatibility examinations. The cells were grown in Dulbecco’s modified Eagle’s medium (DMEM; Sigma-Aldrich, St. Louis, MO, USA) supplemented with 10% (*v/v)* fetal bovine serum (FBS; GIBCO, Franklin Lakes, NJ, USA) and 1.5 g/L sodium bicarbonate. The cells were maintained at 37 °C in an incubator with a 5% CO_2_/95% air atmosphere at constant humidity.

Caco-2 cells were also purchased from Bio-resource Collection and Research Center. They were used in the iron uptake experiment at passage 25 to 35. Cells were seeded at a density of 5 × 10^4^ cells/cm^2^ in 6-well plates. The cells were grown in DMEM (GIBCO) supplemented with 10% (*v/v*) FBS (GIBCO), 1.0 mM sodium pyruvate (P5280; Sigma-Aldrich, St. Louis, MO, USA), 0.01 mg/mL holo-human transferrin (T0665; Sigma-Aldrich, St. Louis, MO, USA), 1% antibiotic alantimycotic solution (GIBCO), and 1.5 g/L sodium bicarbonate. The cells were maintained at 37 °C in the aforementioned CO_2_/air incubator at constant humidity. The medium was changed every second day. The cells were used in the iron uptake experiments 14 days after seeding. At that time, the growth medium was removed from each culture well and the cell layer was washed with PBS at pH 7 before the intestinal digestion period. The supplemented DMEM also contained 10 mM PIPES, 1% antibiotic solution (A5955; Sigma-Aldrich, St. Louis, MO, USA), 4 mg/L hydrocortisone(H0888; Sigma-Aldrich, St. Louis, MO, USA), 5 mg/L insulin (I1882; Sigma-Aldrich, St. Louis, MO, USA), 5 mg/L selenium (S9133; Sigma-Aldrich, St. Louis, MO, USA), 34 mg/L triiodothyronine (T5516; Sigma-Aldrich, St. Louis, MO, USA), and 20 mg/L epidermal growth factor.

### 4.4. Evaluation of L929 Cell Viability

Cell viability following the exposure to MIPs followed the ISO-10993 standard [20]. Briefly, L929 cells (2000 cells/well) were seeded on a 96-well culture dish for 24 h. The extraction solution (0.2 g/mL) were prepared by PBS for 24 h at 37 °C. After 24 h, the L929 culture dish removed supernatants. Then, 100 μL of the experiment extracts solution (the extraction solution and fresh medium were mixed in the ratio of 1:9) was added into a 96-wells culture dish and incubated for 24 h. After that, 100 μL of WST-1 (water-soluble tetrazolium salt; Takara, Japan) solution was added to each well and incubated for 1 h. Using an ELISA reader, the absorbance values of each well were measured at the wavelength of 450 nm.

### 4.5. Immunostaining of Caco-2 Monolayer Tight Junction Structure

To examine the immunocytochemistry of tight junction proteins, we used the staining of washed Caco-2 cell monolayers for the tight junction protein (ZO-1) after being cultured for 14 days. The cells were fixed and permeabilized 400 µL/well fixed solution (methanol: acetone = 1:1) for 20 min at 4 °C. For ZO-1 staining, after washes of the fixed cell dish by PBS, 100 µL/well of primary antibody (1 µg/mL, purified mouse anti-human ZO-1 (BD Transduction Laboratories, Franklin Lakes, NJ, USA) was added and incubated at 4 °C overnight. The cells were washed with PBS, and 100 µL/well of secondary antibody (1 µg/mL Alex 488-conjugated goat anti-rabbit; Invitrogen Pty. Ltd., Mount Waverley VIC, Australia) was added for incubating for 1 h at 37 °C in a cell culture chamber. The cells were washed by PBS. For nuclear staining, 100 µL/well of Hoechst solution (1 µg/mL, Molecular Probes, Eugene, OR, USA) was added each well and incubated in the dark for 5 min at 25 °C. The cells were washed by PBS, the coverslips were mounted using Aqua Polymount (Polysciences, Inc., Warrington, PE, USA) in the dark. The slides were stored in the dark until they were examined under fluorescence microscope (BX51, Olympus, Tokyo, Japan).

### 4.6. Ferritin and Total Protein Analyses of Caco-2 Cell Monolayers

After a 22 h treatment, the culture medium was removed and each cell monolayer washed with 2 mL of rinse solution (140 mM NaCl, 5 mM KCl, and 10 mM PIPES, pH 7.0). The rinse solution was aspirated and 2 mL of removal solution (140 mM NaCl, 5 mM KCl, 10 mM PIPES, 5 mM sodium hydrosulfite, and 1 mM bathophenanthrolene disulfonic acid) was placed for 10 min. This solution was then removed and the cells were lysed by scraping in 50 µL lysis buffer (RIPA lysis buffer; Millipore, Billerica, MA, USA) and a protease inhibitor cocktail (Abcam, Cambridge, UK). Cell lysates were kept on ice for 5 min and then the cells were scraped into an Eppendorf tube. The supernatant containing the proteins was analyzed for ferritin using the Human Ferritin ELISA Kit (Abcam). The ferritin concentration in the samples was determined using a microplate reader at an excitation wavelength of 450 nm according to the manufacturer’s protocol. Ferritin concentrations were normalized to total protein using a protein assay (Bio-Rad, Hercules, CA, USA).

### 4.7. In Vitro Intestinal Digestion

The intestinal digestion was performed as previously described [24]. The iron sample (1.0 mg/mL in deionized water) was used as the stock solution. It was used in 10 mL, 120 mM NaCl, and 5 mM KCl for the simulated digestion. The solution was adjusted to pH 2.0 with 5.0 M HCl in a 50 mL culture tube. To simulate gastric conditions, 0.5 mL pepsin (40 mg/mL (P7012, Sigma-Aldrich) in 0.1 M HCl) was added and the samples were incubated for 60 min on an oscillator at 37 °C. After 60 min, the pH of the samples was gradually adjusted to pH 6.0 with 0.1 M NaHCO_3_ with the immediate addition of 2.5 mL of pancreatin-bile extract (2 mg/mL pancreatin (P3292, Sigma-Aldrich) and 12 mg/mL bile extract (B8631, Sigma-Aldrich) in 0.1 M NaHCO_3_). The samples were further readjusted to pH 7 and the volume was brought up to 15 mL.

A fresh 1.0 mL of supplemented DMEM was used to cover the monolayer of Caco-2 cells. Intestinal lysate (1.5 mL) was placed on top of an upper chamber consisting of a Transwell insert fitted with a 12 kD molecular weight cut-off dialysis membrane (Spectra/Por 7 dialysis tubing, Spectrum Laboratories, Piscataway, NJ, USA) suspended over Caco-2 cell monolayers grown in 6-well plates. The digests were incubated with the cells for 2 h at 37 °C in a humidified incubator containing 5% CO_2_ and 95% air. After 2 h, the inserts were removed, 1 mL of supplemented MEM was added, and cells were incubated for a further 22 h prior to harvesting for ferritin analysis.

### 4.8. In Vivo of Iron Bioavailability in Iron Deficiency Anemia Rats

#### 4.8.1. Iron Deficiency Anemia Rat Model

The availability of iron from MIPs was evaluated in a rat hemoglobin regeneration bioassay before its novel use as an iron source. According to the AOAC standard experimental procedure, rats were fed in an iron depletion period and the iron repletion period stages [25]. Weanling male Wistar rats (Lasco, Taipei, Nangang, Taiwan) weighing 50 ± 5 g were housed individually in stainless-steel cages. The temperature was maintained at 25 °C with 12 h light/dark periods. Food and deionized water were freely available. Each rat was fed an iron-deficient diet (AIN-93G; Research Diets, New Brunswick, NJ, USA) for 21 days (depletion period), the composites are shown in Table 6. The hemoglobin concentration was monitored in blood drawn from the tail. After the 21 day depletion period, the rats were organized into four groups. The rat diet contained supplemental 24 mg/kg elemental iron. To achieve the desired iron, the rats needed to eat 6 g/day of the diet (0.144 mg/day elemental iron). In the repletion period, rats consumed the iron-deficient diet, iron-deficient diet along with 0.72 mg FS per day (FeSO_4_·7H_2_O; F8633, Sigma-Aldrich), iron-deficient diet along with 0.144 mg per day of commercial IP (209309, Sigma-Aldrich), or 0.144 mg per day oral MIPs. Each treatment was carried out for 14 days. All animal experiments were performed according to the guidelines determined by the Animal Center of National Taiwan University for Animal Welfare (IACUC Approval code 20130429, approved on 8 January 2014). All experiments involving animals were carried out at the Animal Center of National Taiwan University.

#### 4.8.2. Iron Bioavailability and Hematological Parameters

Following overnight fasting, Wistar rats were sacrificed by carbon dioxide euthanasia. The blood samples were collected by cardiac puncture and divided into two vials: without anticoagulant for hematology determinations and with anticoagulant (EDTA) for serum biochemical examinations. Hemoglobin gain (Hb) was calculated as the difference in Hb concentration obtained in the final and initial phases of the repletion period. Blood was withdrawn using the intracardiac method, and serum was prepared for the analysis of hematological parameters by the automated hematology analyzer (IDEXX Procyte Dx, Westbrook, MA, USA). The Hb concentrations and Fe consumption results were used to estimate the following parameter:

Hb-Fe was calculated, assuming the total blood volume of 6.7% body weight and hemoglobin iron of 0.335% [29] (Equation (1)):

(1) Hb-Fe(mg)=[body weight (g)×Hb (g/L)×6.7×0.335]10000

Hb regeneration efficiency (HRE) and relative biological value (RBV) were estimated as

(2) $$\%\mathrm{HRE}=\left({\mathrm{Hb}-\mathrm{Fe}}_{\mathrm{Final}}-{\mathrm{Hb}-\mathrm{Fe}}_{\mathrm{Initial}}\right)/{\mathrm{Fe}}_{\mathrm{intake}}\;\left(\mathrm{mg}\right)$$

(3) $$\mathrm{RBV}=100\times \left(\mathrm{HRE}\;\left(\%\right)\;\mathrm{test}\;\mathrm{group}/\mathrm{HRE}\;\left(\%\right)\;{\mathrm{FeSO}}_{4}\cdot 7H_{2}O\;\mathrm{group}\right)$$

### 4.9. In Vivo Study of Subacute Oral Toxicity

#### 4.9.1. Animals

Adult male Wistar rats (Lasco, Taipei, Nangang, Taiwan) at 8 weeks of age were housed individually in stainless-steel cages. The temperature was maintained at 25 °C with 12 h light/dark periods. Food and deionized water were freely available. The animals were divided into two groups: control group (*n* = 10) and mesoporous iron particles group (*n* = 10). Both were administered an oral gavage daily for 28 consecutive days. According to the Daily National Nutritional Ingestion Suggestion Scale, an adult human consumes 15 mg/day of iron. The dosage of an average 70 kg adult requires the conversion of 15 mg/day to an average of 250 g of rats consuming 0.066 mg/day. The control group received only the vehicle (normal saline). Another group received the dosage of MIPs (normal saline + 0.066 mg MIPs) for 28 days. During treatment, every 3 days body weight was determined and food and water consumption, and possible signs of toxicity were observed and recorded, with care taken to treat the rats humanly. At the end of the observation period, all animals were sacrificed by carbon dioxide inhalation and divided into two analysis groups. One group lacked anticoagulant for hematology determination and the other group contained anticoagulant (EDTA) for serum biochemical determinations. All animal experiments were performed according to the guidelines determined by the Animal Center of National Taiwan University for Animal Welfare (IACUC Approval code 20130429, approved on 8 January 2014). All experiments involving animals were carried out at the Animal Center of National Taiwan University.

#### 4.9.2. Hematological, Serum Biochemical, and Histological Analyses

The hematological parameters were determined using an automated hematology analyzer IDEXX ProCyte Dx (IDEXX Laboratories, USA). The total leukocyte count, differential count, counts of erythrocytes and platelets, levels of hemoglobin and hematocrit, and red cell size distribution were determined. Serum biochemical parameters included direct/total bilirubin, cholesterol, electrolytes (potassium, and calcium), markers of renal function (blood urea nitrogen and creatinine), liver markers (alanine aminotransferase and aspartate aminotransferase), and protein profile (albumin, total protein, and globulin). All the determinations were done using Cobas c111 (Roche, USA) using commercial kits (Roche, Basel, Switzerland). After collecting blood, the vital organs (heart, liver, spleen, lung, and kidney) were fixed in 10% buffered formalin, embedded in paraffin, sectioned at 5 μm thickness, stained with H&E, and examined by light microscopy. Histological analysis aimed to assess tissue integrity of the organs.

### 4.10. Statistical Analysis

All experiment results were collected and expressed as mean ± standard deviation (SD). In this study, the statistical analyses were performed using GraphPad Prism 6 (GraphPad Software, San Diego, CA, USA). The result data was considered statistically significant when the *p*-value < 0.05: * *p* < 0.05; ** *p* < 0.01.

## 5. Conclusions

MIPs were successfully synthesized using a modified borohydride reducing method producing iron nanoparticles. The method enabled pronounced porosity and surface area, which increased dissolution. In vitro, the MIPs displayed good biocompatibility. The Caco-2 monolayer model revealed superior bioavailability of the MIPs compared to commercial iron particles. The 28 day oral subacute toxicity experiment revealed no changes in clinical signs, body weight, hematology, blood biochemistry, and histological parameters that could be attributed to the oral administration with MIPs and other agents. In vivo results indicated that MIPs could be suitable as an iron supplement to increase the hemoglobin content of the blood.

## Figures and Tables

**Table 1:** Hematology values of rats after 28 day sub-acute oral toxicity experiment with MIPs.

	Reference [21]	CTL	MIP
WBC (K/uL)	6.5 ± 4.5	11.94 ± 0.79	9.67 ± 2.96
RBC (M/uL)	8.8 ± 1.2	7.79 ± 0.45	7.15 ± 0.52
Hb (g/dL)	13.8 ± 2.3	14.77 ± 0.67	14.63 ± 0.81
HCT (%)	45.3 ± 6.8	43.77 ± 2.44	41.9 ± 2.74
MCV (fL)	51.3 ± 5	56.22 ± 2.4	58.7 ± 2.29
MCH (pg)	17.9 ± 1.6	18.97 ± 0.55	20.51 ± 1.1
MCHC (g/dL)	35.2 ± 3.3	33.75 ± 0.55	34.93 ± 0.99
PLT (K/uL)	913.5 ± 339.5	943.67 ± 187.13	919.5 ± 116.76

CTL: control; MIP: mesoporous iron particles; WBC: white blood cell count; RBC: red blood cell count; Hb: hemoglobin; HCT: hematocrit; MCV: mean corpuscular volume; MCH: mean corpuscular hemoglobin; MCHC: mean cell hemoglobin concentration and PLT: blood platelet count. All data are presented as mean ± SD (*n* = 10).

**Table 2:** Biochemical values of rats after 28 day sub-acute oral toxicity experiment with MIPs.

		Reference [21]	CTL	MIP
ALT/GPT (U/L)		33.5 ± 14.5	35.5 ± 3.64	37.75 ± 4.15
AST /GOT (U/L)		119 ± 56	88.08 ± 10.3	79.45 ± 7.11
ALP (U/L)		83.5 ± 47.5	118.25 ± 15.71	99 ± 19.3
T-Pro (g/dL)		6.6 ± 1	5.6 ± 0.07	5.95 ± 0.25
ALB (g/L)		4.2 ± 0.5	3.87 ± 0.07	4.11 ± 0.18
BUN (mg/dL)		18 ± 3	19.15 ± 1.65	20.36 ± 1.33
Crea (mg/dL)		0.4 ± 0.1	0.24 ± 0.03	0.26 ± 0.05
T-Cho (mg/dL)		66 ± 29	75.08 ± 9.83	72.08 ± 2.72
TG (mg/dL)		93.5 ± 66.5	56.74 ± 7.36	84.77 ± 24.18
AMY (U/L)		1666 ± 443	1658.7 ± 299.97	1905.3 ± 251.45
Glu (mg/dL)		145 ± 39	169.53 ± 35.97	193.96 ± 19.39
Ca (mmol/L)		10.5 ± 1.4	9.93 ± 0.44	9.67 ± 0.13
IP (mg/dL)		6 ± 2.4	8.63 ± 0.45	8.63 ± 0.57

CTL: control; MIP: mesoporous iron particles; AST: aspartate aminotransferase; ALT: alanine aminotransferase; ALP: alkaline phosphatase; T-Pro: total proteins; ALB: albumin; BUN: blood urea nitrogen; Crea: creatinine; T-cho: total cholesterol; TG: triglycerides; AMY: amylase; Glu: glucose; Ca: calcium; and IP: phosphorus. All data are presented as mean ± SD (*n* = 10).

**Table 3:** Hematology values of iron supplements after 14 day repletion in rats.

	Reference [21]	Blank	CTL(+)	FS	IP	MIP
WBC (K/uL)	6.5 ± 4.5	7.22 ± 2.07	4.31 ± 0.77	5.69 ± 3.3	7.76 ± 3.23	5.62 ± 1.72
RBC (M/uL)	8.8 ± 1.2	8.17 ± 0.43	6.54 ± 0.07	8.53 ± 0.49 **	8.62 ± 0.98 *	8.56 ± 0.55 **
Hb (g/dL)	13.8 ± 2.3	15.9 ± 0.72	9.1 ± 0.12	13.75 ± 0.86 **	11.5 ± 0.39 **	12.78 ± 0.81 **
HCT (%)	45.3 ± 6.8	54.67 ± 3.1	28.1 ± 1.62	46.23 ± 3.63 **	40.82 ± 6.59 *	41.8 ± 3.44 **
MCV (fL)	51.3 ± 5	66.94 ± 1.37	42.95 ± 2.02	54.21 ± 3.07 *	47.12 ± 2.67	48.93 ± 4.07 *
MCH (pg)	17.9 ± 1.6	19.48 ± 0.33	13.9 ± 0	16.14 ± 0.7 *	14.24 ± 0.32	14.96 ± 0.88
MCHC (g/dL)	35.2 ± 3.3	29.1 ± 0.34	32.45 ± 1.44	29.75 ± 0.48 *	30.26 ± 1.21	30.63 ± 0.96 *
PLT (K/uL)	913.5 ± 339.5	1122.67 ± 108.13	975.5 ± 307.73	914.25 ± 60.05	1097.2 ± 77.74	1007.5 ± 190.49

Blank: normal diet; CTL(+): AIN-93G diet; FS: AIN-93G + 24 mg/kg FeSO_4_·7H_2_O; IP: AIN-93G + 24 mg/kg commercial iron particles; MIP: AIN-93G + 24 mg/kg meso-iron particles. WBC: white blood cell count; RBC: red blood cell count; Hb: hemoglobin; HCT: hematocrit; MCV: mean corpuscular volume; MCH: mean corpuscular hemoglobin; MCHC: mean cell hemoglobin concentration and PLT: blood platelet count. All data are presented as mean ± SD (*n* = 6). The result compared with standard reference; * *p* < 0.05, ** *p* < 0.01.

**Table 4:** Biochemical values of iron supplements in the 14 day repletion in rats.

	Reference [21]	Blank	CTL(+)	FS	IP	MIP
ALT/GPT (U/L)	33.5 ± 14.5	38.5 ± 2.12	27.06 ± 1.49	45.5 ± 10.97	24.33 ± 0.52	28.33 ± 1.15
AST /GOT (U/L)	119 ± 56	111.4 ± 6.79	146 ± 8.06	174.05 ± 34.01	116.27 ± 13.15	130.37 ± 8.42
ALP (U/L)	83.5 ± 47.5	156.5 ± 9.19	197.7 ± 10.88	206.5 ± 10.97	169 ± 1.79	184.33 ± 15.01
T-Pro (g/dL)	6.6 ± 1	6.55 ± 0.35	6.15 ± 0.35	6.25 ± 0.06	6.07 ± 0.14	6.03 ± 0.15
BUN (mg/dL)	18 ± 3	17.35 ± 1.05	12.7 ± 0.69	25.88 ± 2.34	11.86 ± 1.08	6.93 ± 0.91
Crea (mg/dL)	0.4 ± 0.1	0.38 ± 0.02	0.43 ± 0.02	0.41 ± 0.03	0.38 ± 0.03	0.3 ± 0.01
T-Cho (mg/dL)	66 ± 29	97.5 ± 5.94	79.95 ± 4.45	91.8 ± 9.01	84.5 ± 9.89	98.77 ± 5.32
TG (mg/dL)	93.5 ± 66.5	94.93 ± 5.78	125.06 ± 6.89	74.8 ± 4.37	81.32 ± 4.66	78.56 ± 5.19
Glu (mg/dL)	145 ± 39	78.02 ± 4.75	138.44 ± 7.62	50.43 ± 3.35	92.63 ± 5.61	114 ± 64.52
Ca (mmol/L)	2.6 ± 0.4	3.28 ± 0.2	3.35 ± 0.18	3.2 ± 0.02	3.17 ± 0.03	3.09 ± 0.05
IP (mg/dL)	6 ± 2.4	13.67 ± 0.83	15.33 ± 0.84	15.47 ± 0.58	13.83 ± 0.87	14.58 ± 0.78

Blank: normal diet; CTL(+): AIN-93G diet; FS: AIN-93G + 24 mg/kg FeSO_4_·7H_2_O; IP: AIN-93G + 24 mg/kg commercial iron particles; MIP: AIN-93G + 24 mg/kg mesoporous iron particles; AST: aspartate aminotransferase; ALT: alanine aminotransferase; ALP: alkaline phosphatase; T-Pro: total proteins; BUN: blood urea nitrogen; Crea: creatinine; T-cho: total cholesterol; TG: triglycerides; Glu: glucose; Ca: calcium and IP: phosphorus. All data are presented as mean ± SD (*n* = 6).

**Table 5:** Hematology values in the 21 day iron depletion experiment in rats.

	Reference [21]	Blank	CTL(+)
RBC (M/uL)	8.8 ± 1.2	7.38 ± 0.03	6.19 ±0.5 **
Hb (g/dL)	13.8 ± 2.3	14.97 ± 0.23	10.26 ± 1.18 **
HCT (%)	45.3 ± 6.8	47.43 ± 0.65	29.9 ± 3.92 **

Blank: normal diet; CTL (+): AIN-93G diet. WBC: white blood cell count; RBC: red blood cell count; Hb: hemoglobin and HCT: hematocrit. All data are presented as mean ± SD (*n* = 6). The result compared with standard reference; ** *p* < 0.01.

**Table 6:** Composite of the iron deficiency diet (AIN-93G).

Ingredient	Diet, g/kg
Casein	200
L-Cystine	3
Corn Starch	397.486
Maltodextrin 10	132
Sucrose	100
Avicel, PH101	50
Soybean Oil	70
t-Butylhydroquinone	0.014
Mineral Mix S18706	35
Vitamin Mix V10037	10
Choline Bitartrate	2.5
Red Dye, FD&C #40	0.05

## References

[1] Abbaspour N., Hurrell R., Kelishadi R. (2014). Review on iron and its importance for human health. J. Res. Med. Sci..

[2] Tsukamoto T., Matsubara T., Akashi Y., Kondo M., Yanagita M. (2016). Annual iron loss associated with hemodialysis. Am. J. Nephrol..

[3] Lopez A., Cacoub P., Macdougall I.C., Peyrin-Biroulet L. (2016). Iron deficiency anaemia. Lancet.

[4] Hurrell R., Egli I. (2010). Iron bioavailability and dietary reference values. Am. J. Clin. Nutr..

[5] Fishbane S., Frei G.I., Maesaka J. (1995). Reduction in recombinant human erythropoietin doses by the use of chronic intravenous iron supplementation. Am. J. Kidney Dis..

[6] Andrews N.C., Levy J.E. (1998). Iron is hot: An update on the pathophysiology of hemochromatosis. Blood.

[7] Kaushansky K., Kipps T.J., runton L.L., Hilal-Dandan R., Knollmann C.B. (2017). Hematopoietic Agents: Growth Factors, Minerals, and Vitamins. Gilman’s: The Pharmacological Basis of Therapeutics.

[8] Van Wyck D.B. (1999). Efficacy and adverse effects of oral iron supplements. Seminars in Dialysis.

[9] Harrison B., Pla G.W., Clark G.A., Fritz J.C. (1976). Selection of iron sources for cereal enrichment. Cereal Chem..

[10] He W.-L., Feng Y., Li X.L., Yang X.E. (2008). Comparison of iron uptake from reduced iron powder and FeSO_4_ using the Caco-2 cell model: Effects of ascorbic acid, phytic acid, and pH. J. Agric. Food Chem..

[11] Sacks P.V., Houchin D.N. (1978). Comparative bioavailability of elemental iron powders for repair of iron deficiency anemia in rats. Studies of efficacy and toxicity of carbonyl iron. Am. J. Clin. Nutr..

[12] Arredondo M., Salvat V., Pizarro F., Olivares M. (2006). Smaller iron particle size improves bioavailability of hydrogen-reduced iron-fortified bread. Nutr. Res..

[13] Pattanayak A., Subramanian A. (2011). Impact of porogens on the pore characteristics of zirconia particles made by polymer-induced colloid aggregation. Int. J. Appl. Ceram. Technol..

[14] Suwanprateeb J., Thammarakcharoen F., Phanphiriya P., Chokevivat W., Suvannapruk W., Chernchujit B. (2014). Preparation and characterizations of antibiotic impregnated microporous nano-hydroxyapatite for osteomyelitis treatment. Biomed. Eng. Appl. Basis Commun..

[15] Rao J.-W., Ouyang L.-Q., Jia X.-L., Quan D.-P., Xu Y.-B. (2011). The fabrication and characterization of 3D porous sericin/fibroin blended scaffolds. Biomed. Eng. Appl. Basis Commun..

[16] Silvestri D., Cristallini C., Domenichini M., Gagliardi M., Giusti P. (2010). Non conventional surface functionalization of porous poly-*ε*-caprolactone scaffolds using bioactive molecularly imprinted nanospheres. Biomed. Eng. Appl. Basis Commun..

[17] Marin E.J., Rojas Ciro Y. (2014). A review of polyvinyl alcohol derivatives: Promising materials for pharmaceutical and biomedical applications. Afr. J. Pharm. Pharmacol..

[18] Păduraru O.M., Ciolacu D., Nicoleta R., Vasile D.C. (2012). Synthesis and characterization of polyvinyl alcohol/cellulose cryogels and their testing as carriers for a bioactive component. Mater. Sci. Eng. C.

[19] Sun Y.-P., Lia X.-Q., Caoa J., Zhang W.-X., Wangb H.P. (2006). Characterization of zero-valent iron nanoparticles. Adv. Colloid Interface Sci..

[20] Wallin R.F., Arscott E. (1998). A practical guide to ISO 10993-5: Cytotoxicity. Med. Device Diagn. Ind..

[21] Clifford C.B., Giknis M.A. (2008). Clinical Laboratory Parameters for Crl: Wi (Han) Rats.

[22] Sing K.S. (1985). Reporting physisorption data for gas/solid systems with special reference to the determination of surface area and porosity (Recommendations 1984). Pure Appl. Chem..

[23] Swain J.H., Newman S.M., Hunt J.R. (2003). Bioavailability of elemental iron powders to rats is less than bakery-grade ferrous sulfate and predicted by iron solubility and particle surface area. J. Nutr..

[24] Glahn R.P., Lee O.A., Yeung A., Goldman M.I., Miller D.D. (1998). Caco-2 cell ferritin formation predicts nonradiolabeled food iron availability in an in vitro digestion/Caco-2 cell culture model. J. Nutr..

[25] Shaw N.-S., Liu Y.-H. (2000). Bioavailability of iron from Purple Laver (*Porphyra* spp.) estimated in a rat hemoglobin regeneration bioassay. J. Agric. Food Chem..

[26] Lynch S.R., Bothwell T. (2007). Sustain Task Force on Iron Powders A comparison of physical properties, screening procedures and a human efficacy trial for predicting the bioavailability of commercial elemental iron powders used for food fortification. Int. J. Vit. Nutr. Res..

[27] Kohgo Y., Ikuta K., Ohtake T., Torimoto Y., Kato J. (2008). Body iron metabolism and pathophysiology of iron overload. Int. J. Hematol..

[28] Yang K.-C., Wang C.-C., Wu C.-C., Hung T.-Y., Chang H.-C., Chang H.-K., Lin F.-H. (2010). Acute and subacute oral toxicity tests of sintered dicalcium pyrophosphate on ovariectomized rats for osteoporosis treatment. Biomed. Eng. Appl. Basis Commun..

[29] Mahoney A.W., VanOrden C.C., Hendricks D.G. (1974). Efficiency of converting food iron into hemoglobin by the anemic rat. Ann. Nutr. Metabol..

